# Health vulnerability to vertical transmission of syphilis: validity of constitutive and operational definitions

**DOI:** 10.1590/0034-7167-2024-0656

**Published:** 2026-10-19

**Authors:** Maria Júlia Alexandrino Oliveira, Maria Adelane Monteiro da Silva, Alice Batista Rodrigues, Raquel Sampaio Florêncio, Mayara Nascimento de Vasconcelos, Andrea Carvalho Araújo Moreira, Ana Jessyca Campos Sousa, Antonia Tainá Bezerra Castro

**Affiliations:** I Universidade Federal do Ceará. Sobral, Ceará, Brazil; II Universidade Estadual Vale do Acaraú. Sobral, Ceará, Brazil; III Universidade Estadual do Ceará. Fortaleza, Ceará, Brazil

**Keywords:** Health Vulnerability, Syphilis, Congenital, Pregnant Women, Infectious Disease Transmission, Vertical, Validation Study., Vulnerabilidad en Salud, Sífilis Congénita, Mujeres Embarazadas, Transmisión Vertical de Enfermedad Infecciosa, Estudio de Validación.

## Abstract

**Objective:**

to develop and analyze the validity evidence of the constitutive and operational definitions of health vulnerability for pregnant women in relation to vertical transmission of syphilis.

**Methods:**

a psychometric study, based on the Health Vulnerability Model, protocols from the Ministry of Health, updating the scoping review and conducting a concept analysis. Content validity evidence was analyzed by ten judges. The Content Validity Ratio was used to select operational definitions with a score equal to or greater than 0.8.

**Results:**

the dimensions were divided into nine subdimensions with 17 subconcepts, highlighting “health-disease situation”, “knowledge”, “attitude”, “self-care”, “lifestyle”, “cultural differences”, “social support”, “situations of sexual, physical, verbal, and psychological violence”, “discrimination”, and “general gender inequalities”.

**Final considerations:**

the definitions obtained good validity evidence indices. Thirty operational definitions were developed for construct assessment.

## RESUMEN

**Objetivo:**

desarrollar y analizar la evidencia de validez de contenido de las definiciones constitutivas y operacionales de la dimensionalidad teórica de vulnerabilidad en la salud de las mujeres embarazadas a la transmisión vertical de la sífilis.

**Métodos:**

un estudio psicométrico, basado en el Modelo de Vulnerabilidad en Salud, protocolos del Ministerio de Salud, actualización de la revisión exploratoria y realización de un análisis conceptual. El análisis de la evidencia de validez de contenido fue realizado por diez jueces. Se utilizó la Razón de Validez de Contenidopara seleccionar definiciones operacionales con una puntuación igual o superior a 0,8.

**Resultados:**

las dimensiones se dividieron en nueve subdimensiones con 17 subconceptos, destacando la “situación de salud-enfermedad”, el “conocimiento”, la “actitud”, el “autocuidado”, el “estilo de vida”, las “diferencias culturales”, el “apoyo social”, las “situaciones de violencia sexual, física, verbal y psicológica”, la “discriminación” y las “desigualdades generales de género”.

**Consideraciones finales:**

las definiciones obtuvieron buenos índices de evidencia de validez. Se desarrollaron treinta definiciones operacionales para evaluar el constructo.

## INTRODUCTION

Caused by *Treponema pallidum*, syphilis is a sexually transmitted infection (STI) that can be passed vertically to the fetus of an infected pregnant woman who is untreated or whose treatment regimen has failed, resulting in adverse events such as miscarriage, prematurity, stillbirth, early or late congenital manifestations, and/or death of newborns^(1)^.

In Brazil, in 2023, 25,002 cases of congenital syphilis were reported, with an incidence rate of 9.9 cases per 1,000 live births and 196 infant deaths, resulting in a specific infant mortality rate for congenital syphilis of 7.7 per 100,000 live births^(2)^. In light of this, it is clear that, despite the strategies outlined by the Ministry of Health for preventing STIs, there are still gaps that hinder the control of these diseases, especially syphilis, which has become a serious public health problem^(3)^.

Congenital syphilis points to deficiencies in the functioning of Primary Health Care (PHC), since it is a condition that can be largely prevented through diagnoses, treatments, and public policies provided by the Brazilian Unified Health System (SUS - *Sistema Único de Saúde*). The fragility of prenatal care indicates poor quality of healthcare services, generating vulnerabilities that exacerbate public health problems^(4)^.

Health vulnerability (HV) is understood as a condition of human life resulting from the dynamic relationship between the social subject and precariousness when agency is not experienced in the context of health. Thus, regarding dimensions and subdimensions related to the social subject element, there may be processes of vulnerability and/or conditions of empowerment that generate permanent or temporary situations of vulnerability^(5)^.

The creation of an instrument to assess the HVs of pregnant women related to vertical transmission of syphilis can support PHC professionals, especially nurses, in recognizing these situations. It is worth highlighting the lack of an assessment instrument in this regard. This could result in significant improvements in the care provided to this group. Therefore, identifying these vulnerabilities in a timely manner is fundamental to ensuring quality prenatal care.

In this regard, developing tools based on scientific evidence, taking into account criteria of efficacy, safety, and effectiveness, becomes essential.

## OBJECTIVE

To develop and analyze the validity evidence of the constitutive and operational definitions of HV for pregnant women in relation to vertical transmission of syphilis.

## METHODS

### Ethical aspects

The study was approved by the *Universidade Estadual Vale do Acaraú* Institutional Review Board, following Resolution 466 of the Brazilian National Health Council.

### Study design

This is a psychometric study, referring to the stages corresponding to the theoretical pole of the construction of health instruments^(6)^. The development of a theoretical pole occurs in the content construction and validity stages. For this study, the development processes of the constitutive and operational definitions were described. It was based on the Health Vulnerability Model^(7)^, the Guide for Investigation Committees on Vertical Transmission of HIV, Syphilis and Hepatitis B and C: guidelines for the establishment and updating of VTIC^(8)^, and the Clinical Protocol and Therapeutic Guidelines for Comprehensive Care for People with Sexually Transmitted Infections^(9)^. The COnsolidated criteria for REporting Qualitative research checklist was adopted to ensure the study rigor and quality^(10)^.

### Data collection and data organization

A structure was developed with dimensions divided into subdimensions, containing its constitutive definition followed by an operational definition (OD). A constitutive definition refers to specific concepts of the theory applied in the study, while ODs correspond to concrete actions, such as behaviors, attitudes, or sensations that manifest the construct^(11)^.

The development of ODs was based on an updated scoping review with a protocol registered on the Open Science Framework platform, under the DOI https://doi.org/10.17605/OSF.IO/JBMER, in addition to conducting an analysis of the concept of pregnant women’s vulnerabilities to vertical transmission of syphilis.

### Data analysis

Subsequently, there was an analysis of the content validity evidence of ODs. This stage was conducted through the assessment of expert judges in the subject matter (congenital syphilis/HV and/or psychometric method studies), selected according to criteria that assign higher scores to those with clinical experience, research and publications on the subject, as well as expertise in the conceptual framework involved and methodological knowledge in the construction of questionnaires and scales^(12)^.

A search was conducted on the *Lattes* Platform for specialists who met the aforementioned criteria and obtained five points or more. Subsequently, an invitation letter was sent to the email addresses of these professionals containing all the information about the study, the Informed Consent Form, and a Google Forms^*®*^ questionnaire for assessing content validity evidence. They were also asked to indicate healthcare professionals with expertise in the areas of interest who could act as content reviewers, thus also employing the “snowball sampling” technique.

The criteria for assessing judges were: (1) clarity of language; (2) theoretical relevance; and (3) practical pertinence. The judge was to answer yes/no for each criterion. They were asked to indicate the need for modifications to ODs. This was followed by a qualitative analysis of suggestions and a statistical analysis, using the Content Validity Ratio (CVR), from which ODs with a score equal to or greater than 0.8 were selected. The collected data were organized in a Microsoft Excel^*®*^ spreadsheet.

The CVR was calculated using a specific table applied within the same program^(13)^. After adapting judges’ suggestions, ODs were submitted to a second round of assessment. In both the first and second rounds of assessment, ODs were analyzed by ten expert judges, and the profiles of experts were tabulated and analyzed.

## RESULTS

The theoretical dimensionality was developed from the Health Vulnerability Model^(7)^, consisting of theoretical dimensions and subdimensions adapted to the context of pregnant women with syphilis (Chart 1).

**Chart 1:** Theoretical dimensions and subdimensions of the health vulnerability construct of pregnant women regarding vertical transmission of syphilis. Sobral, Ceará, Brazil, 2024

THEORETICAL DIMENSIONS AND SUBDIMENSIONS OF PREGNANT WOMEN’S VULNERABILITY TO VERTICAL TRANSMISSION OF SYPHILIS
**Subject:** the physical conditions, behaviors, and attitudes of pregnant women with syphilis, along with their interpersonal relationships, influence aspects of knowledge and power, either enhancing or weakening the situations they experience.
**Physical condition:** aspects of a pregnant woman with syphilis in relation to her physical, physiological, or pathological attributes **Functional literacy:** the ability of pregnant women with syphilis to access, understand, analyze, and use general information in order to make decisions and judgments in daily life, acquired through learning, cognition, knowledge, and educational level. **Behavior:** the attitudes of pregnant women with syphilis and their sexual partners related to their self-care and that of the fetus, involving daily care practices as well as their lifestyle. **Interpersonal relationships:** the interaction of pregnant women with syphilis and other people, in response to different forms of power in family, professional, friendship, and affective-sexual relationship contexts.
**Social:** this context is marked by factors that directly influence the actions and social interactions of pregnant women with syphilis, whether with other individuals or healthcare institutions, expanding or limiting their space for self-expression and recognition, both by themselves and by others.
**Socioeconomic situation:** a set of economic, social, educational, and labor-related factors that define a pregnant woman with syphilis or a specific group within a social hierarchy. **Culture:** the ways in which a pregnant woman with syphilis expresses her customs and traditions, passed down and transformed from generation to generation. **Social networks and support:** a group of individuals, family members, or entities, interconnected by different types of relationships, with the purpose of providing emotional, financial, and healthcare support to pregnant women with syphilis. **Violence:** the deliberate use of insults, physical force, or power, whether through threats or concrete actions, against a pregnant woman with syphilis, that results or may result in injury, death, sexual or psychological harm, deprivation for the woman, and/or affects the development of the fetus. **Gender:** it is a sociocultural construct that involves identity behaviors, going beyond biological sex.

*Source: adapted from Florêncio and Moreira^(7)^.*

The dimensions were divided into nine subdimensions, with 17 subconcepts, according to the Health Vulnerability Model. Following this, the scoping review was updated in conjunction with the concept analysis, and three meetings were held between the four authors of this study, during which 30 constitutive and operational definitions were constructed: 20 from the subject dimension and ten from the social dimension (Chart 2).

**Chart 2:** Constitutive and operational definitions of health vulnerability in pregnant women for vertical transmission of syphilis. Sobral, Ceará, Brazil, 2024

SUBCONCEPT	CONSTITUTIVE DEFINITION	OPERATIONAL DEFINITIONS
**SUBJECT**		
Age	Female aged between 13 and 29 years old	Being a young woman (aged between 13 and 29 years old)
Race/color	Self-declaration by a woman of not belonging to the white race	Being a woman of color/race that is not white
Health-illness situation	Pregnancy where the woman does not want or cannot continue the pregnancy	Unwanted pregnancy
	Woman who has had at least one abortion	History of abortion
	Woman diagnosed with a sexually transmitted infection at least once	History of sexually transmitted infections
	Woman previously diagnosed with syphilis	History of syphilis
Education	Woman with incomplete primary education (nine years of education)	Having a low level of education (incomplete primary education)
Knowledge	Lack of reliable information about what a sexually transmitted infection/syphilis is, the complications for the mother and fetus, its forms of prevention, diagnosis, transmission, and treatment	Having limited knowledge about syphilis (prevention, diagnosis, transmission, risks, and treatment)
Attitude	Women who begin their sexual life between the ages of 10 and 19	Having early sexual activity (between 10 and 19 years old)
	Not using a condom in all sexual encounters	Having low adherence to condom use
Self-care	The lack of, refusal of, or abandonment of syphilis treatment associated with non-acceptance of the therapeutic proposal.	Low adherence to syphilis treatment in pregnant women/absence of syphilis treatment in pregnant women/inadequate or incomplete treatment of syphilis in pregnant women
	Sexual partners not undergoing syphilis treatment or with incomplete treatment.	Low adherence to syphilis treatment in sexual partners/absence of syphilis treatment in sexual partners/inadequate or incomplete treatment of pregnant women’s sexual partners with syphilis
Lifestyle	Use of psychoactive substances such as alcohol, cigarettes, or other drugs during pregnancy and lactation	Using psychoactive substances (alcohol, cigarettes, or other drugs) while pregnant
Affective-sexual relationship	Woman without a stable partner during pregnancy	Having multiple sexual partners
	Monogamous relationship without condom use	Having a stable (fixed) sexual partner without using condoms
	Woman who engages in sexual relations in exchange for favors or money, constituting a commercial sexual relationship	Being a sex worker
**SOCIAL**		
Income	Monthly family income of up to half the minimum wage per person residing in the pregnant woman’s household	Having a low family income
Housing	Pregnant women without housing, experiencing homelessness or living in shelters, living with family or friends, without a fixed residence, or living in unsanitary conditions	Having an unfavorable housing situation
Deprivation of liberty	Deprivation of liberty of women and/or sexual partners during pregnancy	Being deprived of liberty during pregnancy/sexual partners in situations of deprivation of liberty
Social constructs	Cultural aspects such as beliefs, religious traditions, forms of communication, and customs that influence practices for preventing vertical transmission	Presenting cultural aspects that influence the prevention of vertical transmission (beliefs, religious practices, forms of communication and customs)
Social support	Unstable ties between family members, which may generate conflicts, lack of communication, misunderstandings, or other factors that compromise harmony and mutual support within the family.	Having fragile family relationships
	Absence or insufficiency of support from the sexual partner in the emotional, financial, and/or prevention/treatment of syphilis and its transmission contexts.	Having ineffective support from a sexual partner (emotional, financial, and in treatment)
Situations of sexual, physical, verbal, and psychological violence	Any situation experienced by a pregnant woman with syphilis that involves an act or intention/threat of violence, whether physical, verbal, psychological, and/or sexual.	Being in a situation of violence (sexual, physical, verbal, and psychological)
Discrimination	Stigma related to the stereotype built around women affected by sexually transmitted infections.	Feeling afraid of the stigma of the disease and of suffering discrimination from society

In the first round of analysis of the content validity evidence of ODs, the panel of judges was predominantly composed of women (90%; n=9), with an average age of 32 years and from the Northeast region (100%; n=10), with nine (90%) residing in Ceará (10%) and one residing in Maranhão. Among judges, seven held a master’s degree and three held a doctoral degree.

All the specialists worked in teaching, and two also in care. Concerning professional training time, all had more than five years of experience, with five professionals having between five and ten years of training, one between 11 and 20 years, and four more than 20 years. Concerning their area of expertise, judges (90%; n=9) have experience in the study of HV, (80%; n=8) have experience in the study of congenital syphilis, and (40%; n=4) have knowledge of the study of psychometric methods.

In relation to the second round of assessment, judges’ characteristics were similar to those of the first, with the majority being female (80%; n=8), with an average age of 29 years, and also from the Northeast region (100%; n=10). Among judges, six held a master’s degree, and four held a doctoral degree. All specialists worked in teaching and two also in clinical practice. Concerning professional training time, two had less than five years, three between six and ten years, and five more than 20 years. Concerning their area of expertise, judges (100%; n=10) had experience in the study of HV, (90%; n=9) had experience in the study of congenital syphilis, and (40%, n=4) had skills in the study of psychometric methods.

Table 1 presents the results of the analysis of the content validity evidence of the ODs of the HV construct to vertical transmission of syphilis, as performed by judges in the first and second rounds of assessment.

**Table 1:** Validity of the content of operational definitions by judges. Sobral, Ceará, Brazil, 2024

	Number of ODs	Operational definitions - 1^st^ round	C	P	R	Number of ODs	Operational definitions - 2^nd^ round	C	P	R
**PHYSICAL CONDITION**	**SUBJECT**									
	1	Young women	0.6	1	1	1	Being a young woman (between the ages of 13 and 29)	1	0.8	1
	2	Woman of non-white color	0.4	1	1	2	Being a woman of non-white race	0.6	1	1
	3	Unwanted pregnancy	1	1	1	3	Unwanted pregnancy	0.8	1	1
	4	History of abortion	1	1	1	4	Presenting a history of abortion	0.6	1	1
	5	History of sexually transmitted infections	1	1	1	5	Presenting a history of STIs	1	1	1
	6	History of syphilis in women	1	1	1	6	Presenting a history of syphilis	0.8	1	1
**FUNCTIONAL LITERACY**	7	Low level of education	0.4	1	1	7	Having low educational attainment (elementary school)	0.6	1	1
	8	Limited knowledge about syphilis	0.2	0.8	1	8	Having little knowledge about syphilis (prevention, diagnosis, transmission, risks and treatment)	0.6	1	1
	9	Limited knowledge about sexual health and sexually transmitted infections	0	1	1					
**BEHAVIOR**	10	Early sexual activity	0.6	1	1	9	Early sexual activity (sexual onset between 10 and 19 years of age)	0.6	1	1
	11	Low adherence to condom use	0.8	1	1	10	Low adherence to condom use	0.6	1	1
	12	Low adherence to syphilis treatment in pregnant women	0.8	1	1	11	Low adherence to syphilis treatment during pregnancy	0.6	1	1
	13	Absence of syphilis treatment in pregnant women	1	1	1	12	Absence of syphilis treatment during pregnancy	0.8	1	1
						13	Inadequate or incomplete treatment of syphilis in pregnant women	0.8	1	1
	14	Poor adherence to syphilis treatment in the partner	0.8	1	1	14	Poor adherence to syphilis treatment in the partner	0.6	1	1
	15	Absence of syphilis treatment in the partner	1	1	1	15	Absence of syphilis treatment in the partner	0.8	1	1
						16	Inadequate or incomplete treatment of pregnant women’s partner with syphilis	0.8	1	1
	16	Use of psychoactive substances by the pregnant woman and/or partner	0.6	0.8	0.8	17	Use of psychoactive substances (alcohol, cigarettes, or other drugs) by the pregnant woman	0.8	1	1
**INTERPERSONAL RELATIONSHIPS**	17	Women with multiple partners	0.8	1	1	18	Having multiple sexual partners	0.8	1	1
	18	Woman in a stable partner not using a condom	0.6	0.8	1	19	Having a stable sexual partner without using a condom	0.8	0.8	0.8
	19	Sex worker women	1	1	1	20	Being a sex worker	0.8	1	1

**SOCIOECONOMIC SITUATION**	**SOCIAL**									
	20	Low socioeconomic status	0.2	1	1					
	21	Low family income	0.4	1	1	21	Having a low family income	0.8	1	1
	22	Unfavorable housing conditions	0.6	1	1	22	Having unfavorable housing conditions	0.8	1	1
	23	Housing in large urban centers	1	1	0.8		Having a low family income			
	24	Housing in rural areas	1	0.8	0.6					
	25	Incarceration of pregnant women or partners	0.6	0.8	0.8	23	Pregnant woman in a situation of deprivation of liberty	1	1	1
						24	Sexual partner in a situation of deprivation of liberty	1	0.8	1
**CULTURA**	26	Cultural aspects	0.2	0.8	0.8	25	Cultural aspects (beliefs, religious practices, forms of communication and customs)	1	1	1
**SOCIAL NETWORKS AND SUPPORTS**	27	Fragile family relationships	1	1	1	26	Having fragile family relationships	0.8	0.8	0.8
	28	Inadequate partner support	0.6	1	1	27	Having ineffective support from sexual partners (emotional, financial, and in treatment)	1	0.8	0.8
**VIOLENCE**	29	Violent situation	0.8	1	1	28	Being in a situation of violence (physical, psychological, sexual, and mental)	0.8	1	1
	30	Stigma and discrimination of the disease	0.6	1	1	29	Feeling fear due to the stigma of the disease and of suffering discrimination from society	0.8	0.8	0.8
**GENDER**	31	Gender-related issues	0	0.8	1	30	Experiencing abusive, dependent, and unequal relationships	0.8	1	1

*Legend: C - clarity; P - pertinence; R - relevance.*

After analyzing the results, adjustments were made to ODs. In the first round, those whose CVR was less than 0.8 in all criteria (clarity, relevance, and pertinence) or in only two of the criteria (relevance and/or pertinence) were excluded. Meanwhile, those that scored less than 0.8 only in the clarity criterion were reformulated according to the suggestions offered by judges.

In this regard, ODs 23 and 24 were excluded due to a CVR of 0.6 in the relevance criterion of OD 24 and because it is understood that it is already included in OD 22 (unfavorable housing conditions). Furthermore, it was decided to exclude OD 20 because its scope is covered by other definitions (income and education).

ODs 1, 2, 7, 8, 9, and 10 obtained a CVR < 0.8 in the clarity criterion. Judge 1 suggested that the ODs were adequate to represent a behavioral trait. Therefore, the suggestion was incorporated for all ODs, as well as for reversing the order of the last two definitions mentioned.

ODs 16 and 25 had a CVR of 0.6 in the clarity criterion. It was suggested that both be split into two definitions to encompass the individual behavior of pregnant women and their partners. For OD 25, it was also suggested that the term “incarceration” be changed to “deprivation of liberty”.

OD 18 received a score of 0.6 on the clarity criterion, raising doubts about the term “stable relationship”, which can be understood in different ways and requires specification. ODs 21 and 22 had CVR scores of 0.4 and 0.6, respectively, on the clarity criterion, with no suggestions from the judges. For OD 26, it was suggested to list the cultural aspects that are intended to be achieved, adopting beliefs, religious practices, forms of communication, and customs, according to the Health Vulnerability Model^(7)^.

OD 28 obtained a CVR of 0.6 in the clarity criterion, indicating the need to specify the type of support being assessed and to change the term “inadequate” to “ineffective”. OD 30 obtained a CVR of 0.6 in the clarity criterion, and it is recommended to clarify the agent who practices discrimination. OD 31 had a CVR of 0 in the clarity criterion, and it is recommended to clarify which gender issues are related to syphilis, given the breadth of the topic involving gender issues. The remaining ODs had a CVR equal to or greater than 0.8 in all assessment criteria.

Judges’ suggestions were accepted. As a result of the adjustments made to ODs, they were submitted to a second validation by judges in order to ensure the effectiveness of the changes made. The data from the 2^nd^ round of validation are presented in Table 1.

Given the new assessment made by judges, it was observed that all ODs passed the criteria of relevance and pertinence. However, ODs 2, 4, 7, 8, 9, 10, 11 and 14 obtained a CVR of 0.6 in the clarity criterion. It was decided not to exclude the ODs with CVR < 0.8 in the clarity criterion, and they were maintained in the dimensionality after undergoing adjustments.

For OD 2, the judge did not consider the term “non-white” clear, and the OD was reformulated. For OD 4, a judge proposed specifying the type of abortion, but the suggestion was not accepted, given that the literature associates syphilis with a greater vulnerability to abortion, without specifying the type. For OD 7, the judge considered the need to delimit the term “elementary education”, modifying it to “incomplete elementary education”. For OD 8, it was suggested to reduce the length of the OD description, but the definition had already been adjusted in the first round, following the guidance of three judges. For OD 9, it was recommended to change the time frame related to the term “precocious”, but the time frame was maintained by literary consensus. For ODs 10 and 11, a judge did not understand what constituted low adherence, which was clarified in the constitutive definition. For OD 14, it was proposed to specify that it refers to sexual partnership. The ODs in their final version after the adjustments are presented in Table 1.

## DISCUSSION

This study involved highly qualified experts. Therefore, the suggestions for improvement justified the decision not to exclude the ODs with unsatisfactory CVR. The same format was used in validation studies in the health field, which adopted a similar methodology, also considering CVR with an index ≥ 0.80 and conducting two rounds of assessment with experts^(14)^.

The constitutive and operational definitions developed in the research, as elements that integrate the theoretical pole of the instrument under construction for assessing HV in pregnant women for vertical transmission of syphilis, comprise the construct dimensions and subdimensions. The listed theoretical dimensions are intended to identify the level of HV and, when associated with their ODs, can express how situations of vulnerability are produced and experienced^(7)^. ODs are essential in research related to the construction of health assessment instruments, as they analyze observable situations in clinical practice to identify HV scenarios^(6)^. During the validation process, some ODs were excluded because they did not obtain agreement among the judges regarding their expression of the latent trait. This reflection of the behavioral paradigm has already been identified in other studies on vulnerability^(15)^.

In the context of congenital syphilis, the pregnant woman emerges as the primary subject, interacting with other individuals and institutions, experiencing vulnerable conditions that can favor vertical transmission of syphilis, but also being capable of preventing it. Hence, the subdimensions adopted for the analysis of the subject element encompass the biological, cognitive-behavioral, and relational fields, such as physical situation, functional literacy, behavior, and interpersonal relationships.

The physical situation refers to the subject’s characteristics in relation to their physical, physiological, or pathological attributes, requiring further investigation into aspects such as age, race/color, and health/illness status^(7)^. It is known that younger women, of non-white race, and with a history of STIs/syphilis and a history of abortion are more vulnerable to vertical transmission of the disease^(8)^.

Functional literacy refers to the knowledge and skills an individual possesses to access, understand, assess, and apply general information in order to make judgments and decisions through learning, cognition, knowledge, and education^(7)^. Pregnant women’s knowledge about syphilis involves understanding its modes of transmission, diagnosis, treatment, and risks to both mother and fetus^(16)^. In this regard, the importance of assessing the level of knowledge of pregnant women as a strategy for preventing gestational and congenital syphilis is highlighted.

The subdimension linked to behavior involves the actions of individuals in relation to their social environment, related to attitudes, self-care or care for others, care practices at work, in routine and daily life, as well as their way of being and communicating^(7)^. The attitude of not using condoms during sexual intercourse is considered a risk factor for syphilis transmission^(9)^. Similarly, inadequate or non-compliance with therapeutic treatment, either by the pregnant woman or her sexual partners, contributes to reinfection and possible vertical transmission^(8)^. It is understood that these behaviors contribute to the vulnerable condition that pregnant women and their partners may be experiencing.

Interpersonal relationships consist of interactions between individuals who communicate verbally or non-verbally, in a movement of responses to certain types of power in situations within the family, at work, in friendships, and in affective-sexual life^(7)^. Thus, it is necessary to assess aspects of the affective-sexual relationship of pregnant women. Women who have had three or more sexual partners within a one-year period have a three times greater risk of syphilis infection^(10)^. Furthermore, women in stable monogamous relationships who do not use condoms due to trust in their partner are also in a vulnerable situation^(17)^. Furthermore, it is important to highlight the context of vulnerability surrounding sex workers, who are exposed to adverse situations ranging from violence to drug use, placing them in a more fragile condition regarding contamination by sexually transmitted infections and their transmission^(18)^.

Social inequalities are structural determinants that directly affect the health of the most vulnerable populations^(19)^. It is agreed that social determinants of health, such as poverty, low educational attainment, and lack of access to quality healthcare services, play a central role in vulnerability to syphilis and its vertical transmission. However, an approach focused solely on structural factors may overlook complex individual and contextual dynamics.

The social context in which a pregnant woman with syphilis is embedded constitutes a space that enables and conditions different interpersonal relationships^(7)^. Therefore, this context influences and is influenced by it, whether in its actions and/or social interactions with other individuals or health institutions and services, which can generate situations of vulnerability and lead to congenital syphilis. Thus, the subdimensions for analyzing the social element involve socioeconomic status, culture, social networks and support, violence, and gender. It is understood that the elements involved in these subdimensions interact with the social determinants in intensifying the conditions of vulnerability.

The incidence of syphilis is higher among pregnant women from unfavorable socioeconomic backgrounds, characterized by poverty, unemployment, and low levels of education^(18)^. In this sense, the components race/color, gender and class are intersected in the context of women with syphilis^(20)^. It is understood that these conditions are the result of socioeconomic and cultural inequalities and structural racism that contribute to the difficulty of access to healthcare services and reliable information, configuring syphilis as a socially determined disease.

Many women live in relationships marked by emotional dependence, permissiveness, conformity, and concealment, losing control over their own bodies/sexuality, placing them in a vulnerable position and making them more exposed to STIs^(21)^. Therefore, paying attention to the context of gender inequalities during the gestational process becomes relevant for syphilis research, especially because it is linked to situations of violence and emotional and psychological distress.

Women who are victims of violence, especially sexual violence, face physical and psycho-emotional vulnerabilities that distance them from healthcare services, hindering early diagnosis of STIs, such as syphilis, or leading to discontinuation of treatment^(22)^. Furthermore, the stigma surrounding STIs in society is based on judgments, stereotypes, and unconscious attitudes that lead to isolation and social exclusion^(23)^.

Thus, it can be observed that the cultural aspects and lifestyles of a given population influence their behavior, actions, and attitudes towards their health-disease-care process. Among these cultural aspects are beliefs, religious practices, forms of communication, and customs^(7)^. Pregnant women who are incarcerated and/or who use psychoactive substances (alcohol, cigarettes, and other drugs) are in a vulnerable situation and, therefore, have a higher chance of transmitting the disease^(8)^. Given these scenarios, it becomes essential to assess the pregnant woman’s attitude towards self-care, her lifestyle, the possibility of adhering to or refusing treatment, as well as the quality of her interpersonal, family, and affective-sexual relationships.

The way women build their emotional and family bonds can influence how they cope with a syphilis diagnosis during pregnancy. In this context, the importance of social networks and support systems is highlighted. These are understood as social structures composed of people or organizations connected by one or more types of relationships, who share common values and objectives and offer support in situations of need for individuals or society^(7)^.

Therefore, it is clear that the risk of vertical transmission of syphilis in pregnant women is a complex and multifactorial condition. However, it is related to a process involving attitudes and behaviors that can be identified through a specific tool, assisting in the planning of interventions that contribute to minimizing the vulnerable context in which pregnant women are inserted, preventing syphilis and its vertical transmission.

### Study limitations

A notable limitation of this study is the scarcity of research linking HV with congenital syphilis, highlighting the novelty of the content presented here. Additionally, there is a lack of clear definitions/explanations in the literature capable of comprehensively conceptualizing the vulnerabilities associated with the phenomenon under study. The difficulty in obtaining responses from the experts contacted for the validity process of the vulnerabilities also constituted a limiting factor.

### Contributions to nursing, health, or public policy

This study allows us to understand the dimensions in which vulnerability can manifest in the lives of pregnant women with syphilis, enabling a more comprehensive and emphatic approach from professionals, especially nurses, in evidence-based care. Furthermore, it will serve as a framework for the subsequent development of an instrument capable of identifying the HVs of pregnant women to vertical transmission of syphilis in their different contexts.

## FINAL CONSIDERATIONS

The definitions obtained good validity evidence indices. The adjustments made in the first round were validated by judges in the second round, and experts’ suggestions improved the instrument. The theoretical dimensionality of the HV applied to vertical transmission of syphilis is composed of two dimensions, nine subdimensions, and 30 constitutive and operational definitions: 22 from the subject dimension and eight from the social dimension. Studies seeking evidence of the validity of the response process and the instrument internal structure are needed to confirm the extent to which the theoretical dimensionality applies to the target population.

## Data Availability

The available research data is in the body of the article.
