# Supplementary material for: Health vulnerability to vertical transmission of syphilis: validity of constitutive and operational definitions
Source: Rev Bras Enferm. 2026 Oct 19;79:e20240656. doi: 10.1590/0034-7167-2024-0656 (PMC13679585; doi:10.1590/0034-7167-2024-0656)
Supplement: Translation

# Translation: Vulnerabilidade em saúde à transmissão vertical da sífilis: validação das definições constitutivas e operacionais

**DOI:** 10.1590/0034-7167-2024-0656pt

**Authors:** Maria Júlia Alexandrino Oliveira, Maria Adelane Monteiro da Silva, Alice Batista Rodrigues, Raquel Sampaio Florêncio, Mayara Nascimento de Vasconcelos, Andrea Carvalho Araújo Moreira, Ana Jessyca Campos Sousa, Antonia Tainá Bezerra Castro

**Affiliations:** I Universidade Federal do Ceará. Sobral, Ceará, Brasil; II Universidade Estadual Vale do Acaraú. Sobral, Ceará, Brasil; III Universidade Estadual do Ceará. Fortaleza, Ceará, Brasil

**Keywords:** Vulnerabilidade em Saúde, Sífilis Congênita, Gestantes, Transmissão Vertical, Estudo de Validação.

## Abstract

**Objetivo:**

elaborar e analisar as evidências de validade de conteúdo das definições constitutivas e operacionais da dimensionalidade teórica da vulnerabilidade em saúde de gestantes à transmissão vertical da sífilis.

**Métodos:**

estudo psicométrico, baseado no Modelo de Vulnerabilidade em Saúde, protocolos do Ministério da Saúde, atualização da revisão de escopo e realização de uma análise do conceito. A análise das evidências de validade de conteúdo foi realizada por dez juízes. Utilizou-se o cálculo da Razão de Validade de Conteúdo para seleção das definições operacionais com pontuação igual ou superior a 0,8.

**Resultados:**

as dimensões foram divididas em nove subdimensões com 17 subconceitos, destacando-se “situação de saúde-doença”, “conhecimento”, “atitude”, “autocuidado”, “estilo de vida”, “diferenças culturais”, “apoio social”, “situações de violência sexual, física, verbal e psicológica”, “discriminação” e “desigualdades gerais de gênero”.

**Considerações finais:**

as definições obtiveram bons índices de evidência de validade. Elaboraram-se 30 definições operacionais para avaliação do construto.

## INTRODUÇÃO

Causada pelo *Treponema pallidum*, a sífilis é uma infecção sexualmente transmissível (IST) que pode passar verticalmente para o concepto da gestante infectada, não tratada ou que apresentou falhas no esquema terapêutico, resultando em eventos adversos como abortamento, prematuridade, natimortalidade, manifestações congênitas precoces ou tardias e/ou morte do recém-nascido^(1)^.

No Brasil, em 2023, foram notificados 25.002 casos de sífilis congênita, além de uma taxa de incidência de 9,9 casos por 1.000 nascidos vivos (NVs) e de 196 óbitos infantis, com coeficiente de mortalidade infantil específica por sífilis congênita de 7,7 por 100.000 NVs^(2)^. Em vista disso, percebe-se que, apesar das estratégias traçadas pelo Ministério da Saúde para prevenção das ISTs, ainda existem lacunas que dificultam o controle dessas doenças, especialmente da sífilis, que vem se configurando como um grave problema de saúde pública^(3)^.

A sífilis congênita aponta para deficiências no funcionamento da Atenção Primária à Saúde (APS), uma vez que é uma condição que pode ser amplamente prevenida por meio de diagnósticos, tratamentos e políticas públicas disponibilizadas pelo Sistema Único de Saúde (SUS). A fragilidade do pré-natal representa um indicativo de má qualidade dos serviços de saúde, gerando vulnerabilidades que acentuam os problemas de saúde pública^(4)^.

Compreende-se a vulnerabilidade em saúde (VS) como uma condição da vida humana resultante da relação dinâmica entre o sujeito-social e que gera precariedade quando o agenciamento não é vivenciado no contexto da saúde. Desse modo, no que tange às dimensões e subdimensões referentes ao elemento sujeito-social, podem existir processos de vulnerabilização e/ou condições de empoderamento que geram situações permanentes ou temporárias de vulnerabilidade^(5)^.

A criação de um instrumento para avaliar as VSs de gestantes relacionadas à transmissão vertical da sífilis pode apoiar os profissionais da APS, especialmente os enfermeiros, no reconhecimento dessas situações. Vale ressaltar a inexistência de um instrumento de avaliação nesse sentido. Isso poderá resultar em melhorias significativas nos cuidados prestados a esse grupo. Portanto, identificar em momento oportuno essas vulnerabilidades é fundamental para garantir um pré-natal de qualidade.

Nesse sentido, desenvolver ferramentas fundamentadas em evidências científicas, levando em conta critérios de eficácia, segurança e efetividade, torna-se essencial.

## OBJETIVO

Elaborar e analisar as evidências de validade das definições constitutivas e operacionais da VS de gestantes à transmissão vertical da sífilis.

## MÉTODOS

### Aspectos éticos

O estudo foi aprovado pelo Comitê de Ética em Pesquisa com Seres Humanos da Universidade Estadual Vale do Acaraú, seguindo a Resolução nº 466 do Conselho Nacional de Saúde.

### Tipo de estudo

Trata-se de estudo psicométrico, referente às etapas correspondentes ao polo teórico da construção de instrumentos em saúde^(6)^. O desenvolvimento do polo teórico se dá nas etapas de construção e validação de conteúdo. Para este estudo, descreveram-se os processos de desenvolvimento das definições constitutivas e operacionais. Fundamentou-se no Modelo de Vulnerabilidade em Saúde^(7)^, no Guia de Comitês de Investigação da Transmissão Vertical de HIV, Sífilis e Hepatites B e C: orientações para instituição e atualização de CITV^(8)^, e no Protocolo Clínico e Diretrizes Terapêuticas para Atenção Integral às Pessoas com Infecções Sexualmente Transmissíveis^(9)^. Adotou-se o *checklist COnsolidated criteria for REporting Qualitative research* para garantir o rigor e a qualidade do estudo^(10)^.

### Coleta de dados e organização dos dados

Elaborou-se uma estrutura com dimensões divididas em subdimensões, contendo sua definição constitutiva seguida de uma definição operacional (DO). A definição constitutiva se refere a conceitos específicos da teoria aplicada no estudo, enquanto as DOs correspondem a ações concretas, como comportamentos, atitudes ou sensações que manifestam o construto^(11)^.

A construção das DOs se deu a partir da atualização da revisão de escopo com protocolo registrado na plataforma *Open Science Framework*, sob o DOI https://doi.org/10.17605/OSF.IO/JBMER, somada à realização de uma análise do conceito das vulnerabilidades de gestante à transmissão vertical da sífilis.

### Análise dos dados

Posteriormente, seguiu-se com a análise das evidências de validade de conteúdo das DOs. Essa etapa foi conduzida pela avaliação de juízes especialistas na temática (sífilis congênita/VS e/ou estudos de método psicométrico), selecionados conforme critérios que atribuem maior pontuação àqueles com experiência clínica, pesquisas e publicações sobre o tema, bem como *expertise* na estrutura conceitual envolvida e conhecimento metodológico na construção de questionários e escalas^(12)^.

Realizou-se uma busca na Plataforma *Lattes* pelos especialistas que atendiam aos critérios mencionados e obtiveram cinco pontos ou mais. Em seguida, enviou-se uma carta-convite ao e-mail desses profissionais contendo todas as informações sobre o estudo, o Termo de Consentimento Livre e Esclarecido e um formulário via *Google Forms^®^* para a avaliação das evidências de validade de conteúdo. Solicitou-se ainda a indicação de profissionais de saúde com *expertise* nas áreas de interesse que pudessem atuar como avaliadores de conteúdo, caracterizando também a utilização da técnica “bola de neve”.

Os critérios para avaliação dos juízes foram: (1) clareza na linguagem; (2) relevância teórica; e (3) pertinência prática. O juiz deveria responder entre sim/não para cada critério. Solicitou-se indicar a necessidade de modificações nas DOs. Seguiu-se com a análise qualitativa das sugestões e análise estatística, e utilizou-se a Razão de Validade de Conteúdo (RVC), a partir do qual foram selecionadas as DOs com pontuação igual ou superior a 0,8. Os dados coletados foram organizados na planilha do *Microsoft Excel^®^*.

O RVC foi calculado em tabela específica aplicada dentro do mesmo programa^(13)^. Após adequação das sugestões dos juízes, as DOs foram submetidas a uma segunda rodada de avaliação. Tanto na primeira quanto na segunda rodada de avaliação, as DOs foram analisadas por dez juízes *experts*, sendo o perfil dos especialistas tabulado e analisado.

## RESULTADOS

A dimensionalidade teórica foi elaborada a partir do Modelo de Vulnerabilidade em Saúde^(7)^, constituído por dimensões e subdimensões teóricas adaptadas para o contexto da gestante com sífilis (Quadro 1).

**Quadro 1:** Dimensões e subdimensões teóricas do construto vulnerabilidade em saúde de gestantes para a transmissão vertical da sífilis. Sobral, Ceará, Brasil, 2024

DIMENSÕES E SUBDIMENSÕES TEÓRICAS DA VULNERABILIDADE DE GESTANTES À TRANSMISSÃO VERTICAL DA SÍFILIS
**Sujeito:** condições físicas, comportamentos e atitudes da gestante com sífilis e suas relações interpessoais que condicionam os aspectos do saber e poder, potencializando ou fragilizando as situações vivenciadas.
**Situação física:** aspectos da gestante com sífilis em relação aos seus atributos físicos, fisiológicos ou patológicos. **Letramento funcional:** a capacidade das gestantes com sífilis de acessar, entender, analisar e utilizar informações gerais, de modo a tomar decisões e fazer julgamentos no cotidiano, adquirida por meio de aprendizado, cognição, conhecimento e nível educacional. **Comportamento:** atitudes da gestante com sífilis e suas parcerias sexuais relacionadas ao seu autocuidado e do concepto, envolvendo as práticas de cuidado cotidianas, bem como seu modo de vida. **Relações interpessoais:** interação da gestante com sífilis com as demais pessoas, em resposta a diferentes formas de poder em contextos familiares, profissionais, de amizade e nas relações afetivo-sexuais.
**Social:** contexto marcado por fatores que influenciam diretamente as ações e interações sociais da gestante com sífilis, seja com outros sujeitos ou instituições de saúde, ampliando ou limitando seu espaço de expressão e reconhecimento por si e pelo outro.
**Situação socioeconômica:** conjunto de aspectos econômicos, sociais, educacionais e laborais que definem a gestante com sífilis ou um grupo específico dentro de uma hierarquia social. **Cultura:** as maneiras pelas quais a gestante com sífilis expressa seus costumes e tradições, transmitidos e transformados de geração em geração. **Redes e suportes sociais:** grupo de indivíduos, familiares ou entidades interligados por diferentes tipos de relações, com o propósito de fornecer apoio tanto emocional quanto financeiro e no cuidado à saúde da gestante com sífilis. **Violência:** o uso deliberado de insultos, força física ou poder, seja por meio de ameaças ou ações concretas, contra uma mulher grávida com sífilis, que resulte ou possa resultar em lesão, morte, dano sexual ou psicológico, privação para a mulher e/ou afetar o desenvolvimento do feto. **Gênero:** trata-se de uma construção sociocultural, que envolve comportamentos identitários, indo além do sexo biológico.

*Fonte: adaptado de Florêncio e Moreira^(7)^.*

As dimensões foram divididas em nove subdimensões, com 17 subconceitos, de acordo com o Modelo de Vulnerabilidade em Saúde. Em seguida, com a atualização da revisão de escopo em conjunto com a análise do conceito, seguida por três reuniões entre quatro autores deste estudo, durante as quais foram construídas 30 definições constitutivas e operacionais: 20 da dimensão sujeito e dez da dimensão social (Quadro 2):

**Quadro 2:** Definições constitutivas e operacionais da vulnerabilidade em saúde de gestantes para a transmissão vertical da sífilis. Sobral, Ceará, Brasil, 2024

SUBCONCEITO	DEFINIÇÃO CONSTITUTIVA	DEFINIÇÕES OPERACIONAIS
**SUJEITO**		
Idade	Mulher com idade entre 13 e 29 anos	Ser mulher jovem(idade entre 13 e 29 anos)
Raça/cor	Autodeclaração da mulher de não pertencimento à raça branca	Ser mulher de cor/raça não branca
Situação de saúde-doença	Gravidez em que a mulher não quer ou não pode levar adiante a gestação	Gravidez indesejada
	Mulher que já abortou pelo menos uma vez	Apresentar histórico de aborto
	Mulher já diagnosticada com infeccção sexualmente transmissível pelo menos uma vez	Apresentar histórico de infecções sexualmente transmissíveis
	Mulher já foi diagnosticada com sífilis anteriormente	Apresentar histórico de sífilis
Escolaridade	Mulher com escolarização incompleta do ensino fundamental de nove anos de estudo	Ter baixa escolaridade (ensino fundamental incompleto)
Conhecimento	Insuficiência de informações seguras sobre do que se trata uma infeccção sexualmente transmissível/sífilis, as complicações para mãe e para o feto, além de suas formas de prevenção, diagnóstico, transmissão e tratamento	Ter pouco conhecimento sobre sífilis (prevenção, diagnóstico, transmissão, riscos e tratamento)
Atitude	Mulher com iniciação da vida sexual entre 10 e 19 anos de idade	Ter prática sexual precoce
	Não utilização do preservativo em todas as relações sexuais	Ter baixa adesão ao uso do preservativo
Autocuidado	A falta, recusa ou abandono do tratamento da sífilis associados à não aceitação da proposta terapêutica	Ter baixa adesão ao tratamento da sífilis na gestante/ausência do tratamento da sífilis na gestante/tratamento inadequado ou incompleto da sífilis na gestante
	Parcerias sexual não submetida ao tratamento da sífilis ou com tratamento incompleto	Ter baixa adesão ao tratamento da sífilis nas parcerias sexuais/ausência do tratamento da sífilis nas parcerias sexuais/tratamento inadequado ou incompleto das parcerias sexuais da gestante com sífilis
Estilo de vida	Uso de substâncias psicoativas como álcool, cigarro ou outras drogas durante a gestação e lactação	Fazer uso de substâncias psicoativas (álcool, cigarro ou outras drogas) pela gestante
Relação afetivo-sexual	Mulher sem uma parceria fixa durante a gestação	Ter múltiplas parcerias sexuais
	Relação monogâmica sem uso de preservativo	Ter parceria sexual estável (fixo), sem uso de preservativo
	Mulher que mantém relação sexual em troca de favores ou dinheiro, constituindo relação sexual comercial	Ser profissional do sexo
**SOCIAL**		
Renda	Renda familiar mensal de até meio salário mínimo por pessoa residente no domicílio da gestante	Ter baixa renda familiar
Moradia	Gestantes sem moradia, em situação de rua ou em albergues, que vivem com familiares ou amigos, sem residência fixa ou vivem em situações insalubres	Ter situação de moradia desfavorável
Privação de liberdade	Privação de liberdade da mulher e/ou da parceria sexual durante a gestação	Estar em situação de privação de liberdade na gestação/parcerias sexuais em situação de privação de liberdade
Construções sociais	Aspectos culturais como crenças, tradições religiosas, formas de comunicação e costumes que influenciam as práticas de prevenção da transmissão vertical	Apresentar aspectos culturais que influenciam a prevenção da transmissão vertical (crenças, práticas religiosas, formas de comunicação e costumes)
Apoio social	Laços instáveis entre os membros da família, podendo gerar conflitos, falta de comunicação, desentendimentos ou outros fatores que comprometem a harmonia e o apoio mútuo dentro da família	Ter relações familiares fragilizadas
	Ausência ou insuficiência de apoio da parceria sexual tanto no contexto emocional quanto no financeiro e/ou para prática de prevenção/tratamento da sífilis e sua transmissão	Ter apoio ineficaz do parceiro sexual (emocional, financeiro e no tratamento)
Situações de violência sexual, física, verbal e psicológica	Qualquer situação vivenciada pela gestante com sífilis que envolva ato ou intenção/ameaça de violência, seja física, verbal, psicológica e/ou sexual	Estar em situação de violência (sexual, física, verbal e psicológica)
Discriminação	Estigma relacionado ao estereótipo construído acerca das mulheres acometidas com infecções sexualmente transmissíveis, discriminação pela sociedade das mulheres acometidas com infecções sexualmente transmissíveis	Sentir medo pelo estigma da doença e de sofrer discriminação pela sociedade

Na primeira rodada de análise das evidências de validade de conteúdo das DOs, o corpo de juízes foi composto predominantemente de mulheres (90%; n=9), com média de idade de 32 anos e da região Nordeste (100%; n=10), sendo nove (90%) residentes do Ceará (10%) e uma residente do Maranhão. Entre os juízes, sete possuíam titulação de mestre e três possuíam de doutor.

Todos os especialistas atuavam na docência e dois também na assistência. Em relação ao tempo de formação profissional, todos possuíam tempo superior a cinco anos, sendo cinco profissionais com formação entre cinco e dez anos, um, entre 11 e 20 anos, e quatro, superior a 20 anos. No tocante à área de *expertise*, os juízes (90%; n=9) possuem experiência no estudo da VS, (80%; n=8) vivência no estudo da sífilis congênita e (40%; n=4) conhecimento do estudo de métodos psicométricos.

Quanto à segunda rodada de avaliação, as características dos juízes foram semelhantes às da primeira, sendo a maioria do sexo feminino (80%; n=8), com média de idade de 29 anos, e eram também da região Nordeste (100%; n=10). Entre os juízes, seis possuíam o título de mestre, e quatro, de doutor. Todos os especialistas atuavam na docência e dois também na assistência. Em relação ao tempo de formação profissional, dois possuíam tempo inferior a cinco anos, três, entre seis e dez anos, e cinco, superior a 20 anos. No tocante a área de *expertise*, os juízes (100%; n=10) possuem experiência no estudo da VS, (90%; n=9) vivência no estudo da sífilis congênita e (40%, n=4) habilidades no estudo de métodos psicométricos.

Na Tabela 1, apresentam-se os resultados da análise das evidências de validade de conteúdo das DOs do construto VS à transmissão vertical da sífilis feita pelos juízes na primeira e segunda rodada de avaliação.

**Tabela 1:** Validação de conteúdo das definições operacionais pelos juízes. Sobral, Ceará, Brasil, 2024

	N^o^ da DO	Definições operacionais - 1ª rodada	C	P	R	N^o^ da DO	Definições operacionais - 2ª rodada	C	P	R
**SITUAÇÃO FÍSICA**	**SUJEITO**									
	1	Mulheres jovens	0,6	1	1	1	Ser mulher jovem (idade entre 13 e 29 anos)	1	0,8	1
	2	Mulher de cor não branca	0,4	1	1	2	Ser mulher de cor não branca	0,6	1	1
	3	Gravidez indesejada	1	1	1	3	Gravidez indesejada	0,8	1	1
	4	Histórico de aborto	1	1	1	4	Apresentar histórico de aborto	0,6	1	1
	5	Histórico de infecções sexualmente transmissíveis	1	1	1	5	Apresentar histórico de ist	1	1	1
	6	Histórico de sífilis na mulher	1	1	1	6	Apresentar histórico de sífilis	0,8	1	1
**LETRAMENTO FUNCIONAL**	7	Baixa escolaridade	0,4	1	1	7	Ter baixa escolaridade (ensino fundamental)	0,6	1	1
	8	Pouco conhecimento sobre sífilis	0,2	0,8	1	8	Ter pouco conhecimento sobre sífilis (prevenção, diagnóstico, transmissão, riscos e tratamento)	0,6	1	1
	9	Pouco conhecimento em saúde sexual e infecções sexualmente transmissíveis	0	1	1					
**COMPORTAMENTO**	10	Prática sexual precoce	0,6	1	1	9	Ter prática sexual precoce (início sexual entre 10 e 19 anos)	0,6	1	1
	11	Baixa adesão ao uso do preservativo	0,8	1	1	10	Ter baixa adesão ao uso do preservativo	0,6	1	1
	12	Baixa adesão ao tratamento da sífilis na gestante	0,8	1	1	11	Ter baixa adesão ao tratamento da sífilis na gestante	0,6	1	1
	13	Ausência do tratamento da sífilis na gestante	1	1	1	12	Ausência do tratamento da sífilis na gestante	0,8	1	1
						13	Tratamento inadequado ou incompleto da sífilis na gestante	0,8	1	1
	14	Baixa adesão ao tratamento da sífilis no parceiro	0,8	1	1	14	Ter baixa adesão ao tratamento da sífilis no parceiro	0,6	1	1
	15	Ausência do tratamento da sífilis no parceiro	1	1	1	15	Ausência do tratamento da sífilis no parceiro	0,8	1	1
						16	Tratamento inadequado ou incompleto do parceiro da gestante com sífilis	0,8	1	1
	16	Uso de substâncias psicoativas pela gestante e/ou parceiro	0,6	0,8	0,8	17	Uso de substâncias psicoativas (álcool, cigarro ou outras drogas) pela gestante	0,8	1	1
**RELAÇÕES INTERPESSOAIS**	17	Mulheres com múltiplos parceiros	0,8	1	1	18	Ter múltiplos parceiros sexuais	0,8	1	1
	18	Mulher com parceria estável sem uso de preservativo	0,6	0,8	1	19	Ter parceria sexual estável, sem uso de preservativo	0,8	0,8	0,8
	19	Mulheres trabalhadoras do sexo	1	1	1	20	Ser profissional do sexo	0,8	1	1

**SITUAÇÃO SOCIOECONÔMICA**	**SOCIAL**									
	20	Baixo nível socioeconômico	0,2	1	1					
	21	Baixa renda familiar	0,4	1	1	21	Ter baixa renda familiar	0,8	1	1
	22	Condições de moradia desfavoráveis	0,6	1	1	22	Ter situação de moradia desfavorável	0,8	1	1
	23	Habitação em grande centros urbanos	1	1	0,8					
	24	Habitação em área rural	1	0,8	0,6					
	25	Encarceramento da gestante ou do parceiro	0,6	0,8	0,8	23	Gestante em situação de privação de liberdade	1	1	1
						24	Parceiro sexual em situação de privação de liberdade	1	0,8	1
**CULTURA**	26	Aspectos culturais	0,2	0,8	0,8	25	Aspectos culturais (crenças, práticas religiosas, formas de comunicação e costumes)	1	1	1
**REDES E SUPORTES SOCIAIS**	27	Relações familiares fragilizadas	1	1	1	26	Ter relações familiares fragilizadas	0,8	0,8	0,8
	28	Suporte inadequado do parceiro	0,6	1	1	27	Ter apoio ineficaz das parcerias sexuais (emocional, financeiro e no tratamento)	1	0,8	0,8
**VIOLÊNCIA**	29	Situação de violência	0,8	1	1	28	Estar em situação de violência (física, psicológica, sexual e mental)	0,8	1	1
	30	Estigma e discriminação da doença	0,6	1	1	29	Sentir medo pelo estigma da doença e de sofrer discriminação pela sociedade	0,8	0,8	0,8
**GÊNERO**	31	Questões relacionadas ao gênero	0	0,8	1	30	Vivenciar relações abusivas, de dependência e desigualdade	0,8	1	1

*Legenda: C- clareza; P - pertinência; R - relevância.*

Após análise dos resultados, procedeu-se às adequações das DOs. Na primeira rodada, excluíram-se aquelas cujo RVC foi inferior a 0,8 em todos os critérios (clareza, relevância e pertinência) ou em apenas dois dos critérios (relevância e/ou pertinência). Enquanto isso, as que apresentaram nota inferior a 0,8 apenas no quesito clareza foram reformuladas conforme as sugestões oferecidas pelos juízes.

Nesse sentido, as DOs 23 e 24 foram excluídas em virtude do RVC de 0,6 no critério de relevância da DO 24 e por entender que já está inserida na DO 22 (condições de moradia desfavoráveis). Ademais, optou-se por excluir a DO 20 em virtude de sua abrangência ser contemplada em outras definições (renda e escolaridade).

As DOs 1, 2, 7, 8 e 9, 10 obtiveram RVC < 0,8 no critério clareza. O juíz 1 sugeriu que as DOs fossem adequadas para representar um traço comportamental. Portanto, a sugestão foi incorporada para todas as DOs, bem como para a inversão na ordem das duas últimas definições citadas.

As DOs 16 e 25 tiveram RVC de 0,6 no critério clareza. Foi sugerido o desmembramento de ambas em duas definições para contemplar o comportamento individual da gestante e do parceiro. Na DO 25, sugeriu-se também a mudança do termo “encarceramento” por “privação de liberdade”.

A DO 18 recebeu 0,6 no critério clareza, gerando dúvidas acerca do termo “relacionamento estável”, que pode ser compreendido de diferentes maneiras, necessitando de uma especificação. As DOs 21 e 22 tiveram RVC de 0,4 e 0,6, respectivamente, no critério de clareza, não havendo sugestões dos juízes. Na DO 26, sugeriu-se elencar os aspectos culturais que se almejam atingir, adotando-se crenças, práticas religiosas, formas de comunicação e costumes, conforme o Modelo de Vulnerabilidade em Saúde^(7)^.

A DO 28 obteve RVC de 0,6 no critério de clareza, indicando-se a especificação do tipo de suporte que está sendo avaliado e a troca do termo “inadequado” para “ineficaz”. A DO 30 obteve RVC de 0,6 no critério clareza, orientando-se esclarecer o agente que pratica a discriminação. A DO 31 teve RVC de 0 no critério clareza, recomendando-se esclarecer quais são as questões de gênero que estão relacionadas à sífilis, haja vista a abrangência da temática envolvendo as questões de gênero. As demais DOs tiveram RVC igual ou superior a 0,8 em todos os critérios de avaliação.

As sugestões dos juízes foram acatadas. Em decorrência dos ajustes realizados nas DOs, foram submetidas a uma segunda validação pelos juízes, a fim de certificar-se da eficácia das mudanças executadas. Os dados da 2ª rodada de validação estão apresentados na Tabela 1.

Diante da nova avaliação feita pelos juízes, percebeu-se que todas as DOs passaram nos critérios de relevância e pertinência. Contudo, as DOs 2, 4, 7, 8, 9, 10, 11 e 14 obtiveram RVC de 0,6 no critério clareza. Optou-se por não excluir as DOs com RVC < 0,8 no critério clareza, sendo as mesmas mantidas na dimensionalidade após passarem por ajustes.

Na DO 2, o juiz não considerou claro o termo “não branca”, sendo a DO reformulada. Na DO 4, um juiz propôs especificar o tipo de aborto, porém a sugestão não foi acatada, tendo em vista que a literatura associa a sífilis a uma maior vulnerabilidade da ocorrência de aborto, sem especificar o tipo. Na DO 7, o juiz considerou a necessidade de delimitar o termo “ensino fundamental”, modificando-se para “ensino fundamental incompleto”. Já na DO 8, indicou-se reduzir a extensão da descrição da DO, porém a definição já havia passado por ajustes na primeira rodada, seguindo a orientação de três juízes. Na DO 9, recomendou-se alterar o tempo referente ao termo “precoce”, mas o tempo foi mantido por consenso literário. Nas DOs 10 e 11, um juiz não compreendeu o que seria a baixa adesão, sendo, assim, esclarecido na definição constitutiva. Na DO 14, foi proposto especificar que se trata da parceria sexual. As DOs na versão final após os ajustes estão apresentadas na Tabela 1.

## DISCUSSÃO

Este estudo contou com especialistas com alto grau de qualificação. Portanto, as sugestões de melhorias fundamentaram a decisão de não excluir as DOs com RVC insatisfatório. O mesmo formato foi utilizado em estudos de validação na área da saúde, que adotaram metodologia semelhante, que consideraram também RVC com índice ≥ 0,80 e realizaram duas rodadas de avaliação com os especialistas^(14)^.

As definições constitutiva e operacionais desenvolvidas na pesquisa, como elementos que integram o polo teórico do instrumento em construção para avaliação da VS em gestantes para a transmissão vertical da sífilis, compõem as dimensões e subdimensões do constructo. As dimensões teóricas elencadas se destinam à identificação do nível de VS e, quando associadas às suas DOs, podem expressar como as situações de vulnerabilidade são produzidas e vivenciadas^(7)^. As DOs são essenciais nas pesquisas relacionadas à construção de instrumentos de avaliação em saúde, pois analisam situações observáveis na prática clínica para identificar cenários de VS^(6)^. Durante o processo de validação, algumas DOs foram excluídas por não terem obtido concordância entre os juízes no que se refere à sua expressão do traço latente. Esse reflexo do paradigma comportamental já foi identificado em outros estudos sobre vulnerabilidade^(15)^.

No cenário que envolve a sífilis congênita, a gestante se apresenta como sujeito principal, em relação com outros sujeitos e instituições, vivenciando condições de vulnerabilidade que podem favorecer a transmissão vertical da sífilis, mas também sendo capaz de preveni-la. Nesse sentido, as subdimensões adotadas para análise do elemento sujeito perpassam os campos do biológico, cognitivo-comportamental e das relações, como situação-física, letramento funcional, comportamento e relações interpessoais.

A situação-física remete às características do sujeito em relação aos seus atributos físicos, fisiológicos ou patológicos, necessitando de maior investigação nos aspectos da idade, raça/cor e situação de saúde-doença^(7)^. Sabe-se que as mulheres mais jovens, de cor não branca e com histórico para IST/sífilis e histórico de aborto apresentam maior vulnerabilidade para transmissão vertical da doença^(8)^.

O letramento funcional corresponde ao conhecimento e às competências do sujeito para acessar, compreender, avaliar e aplicar informações gerais, de forma a fazer julgamentos e tomar decisões por meio da aprendizagem, cognição, conhecimento e escolaridade^(7)^. O conhecimento das gestantes sobre sífilis envolve a compreensão de suas formas de transmissão, diagnóstico, tratamento, e riscos para a mãe e para o concepto^(16)^. Nesse sentido, destaca-se a importância da avaliação do nível de conhecimento das gestantes como estratégia para prevenção de sífilis gestacional e congênita.

A subdimensão vinculada ao comportamento envolve as ações dos sujeitos em relação ao seu meio social, relacionadas às atitudes, ao autocuidado ou ao cuidado de outrem, às práticas de cuidado no trabalho, na rotina e no cotidiano, bem como ao modo de ser e de comunicação^(7)^. Considera-se que a atitude da não utilização de preservativo durante a prática sexual é considerada um fator de risco para a transmissão sífilis^(9)^. Do mesmo modo, a realização inadequada ou não realização do tratamento terapêutico, seja pela gestante ou por suas parcerias sexuais, corrobora a reinfecção e possível transmissão vertical^(8)^. Entende-se que esses comportamentos corroboram para a condição de vulnerabilização em que gestantes e parcerias possam estão vivenciando.

As relações interpessoais constituem-se nas interações entre sujeitos que se comunicam verbal ou não verbalmente, em um movimento de respostas a determinados tipos de poder em situações na família, no trabalho, na amizade e na vida afetiva-sexual^(7)^. Assim, observa-se a necessidade de avaliar os aspectos da relação afetivo-sexual das gestantes. Mulheres que tiveram três ou mais parceiros sexuais em um intervalo de um ano possuem risco três vezes maior de infecção por sífilis^(10)^. Ademais, mulheres em relação monogâmica estável que não fazem o uso de preservativos devido à confiança no parceiro também se encontram em situação de vulnerabilidade^(17)^. Ressalta-se, ainda, o contexto de vulnerabilização que envolve as trabalhadoras do sexo, expostas a situações adversas, que vão desde a violência ao uso de drogas, colocando-as em uma condição de maior fragilidade para a contaminação por infecções sexualmente transmissíveis e sua transmissão^(18)^.

As desigualdades sociais são determinantes estruturais que afetam diretamente a saúde das populações mais vulneráveis^(19)^. Concorda-se que os determinantes sociais de saúde, como pobreza, baixo nível educacional e falta de acesso a serviços de saúde de qualidade, têm papel central na vulnerabilidade à sífilis e sua transmissão vertical. No entanto, uma abordagem com foco apenas em fatores estruturais pode negligenciar as complexas dinâmicas individuais e contextuais.

O contexto social no qual a gestante com sífilis está inserida constitui espaço que possibilita e condiciona as diferentes relações interpessoais^(7)^. Logo, esse contexto influencia e é influenciado por ela, seja nas suas ações e/ou interações sociais com outros sujeitos ou instituições e serviços de saúde, que podem gerar situações de vulnerabilidades e que podem levar à sífilis congênita. Desse modo, as subdimensões para análise do elemento social envolvem situação socioeconômica, cultura, redes e suportes sociais, violência, e gênero. Entende-se que os elementos que envolvem essas subdimensões interagem com os determinantes sociais na intensificação das condições de vulnerabilização.

A incidência de sífilis é maior entre as gestantes com estrutura socioeconômica desfavorável, marcada pela pobreza, pelo desemprego e pela baixa escolaridade^(18)^. Nesse sentido, os componentes raça/cor, gênero e classe se apresentam de forma interseccionada no contexto da mulher com sífilis^(20)^. Entende-se que essas condições são resultantes das desigualdades socioeconômicas, culturais e do racismo estrutural, que contribuem para a dificuldade de acesso aos serviços de saúde e à informação segura, configurando a sífilis como uma doença socialmente determinada.

Muitas mulheres vivem relações marcadas pela dependência afetiva, permissividade, conformismo e ocultamento, perdendo o controle sobre o próprio corpo/sexualidade, colocando-as em condição de vulnerabilização e mais expostas às ISTs^(21)^. Logo, atentar-se para o contexto das desigualdades de gênero durante o processo gestacional é relevante para a investigação da sífilis, especialmente por estar atrelado às situações de violência e ao sofrimento emocional e psíquico.

Mulheres vítimas de violência, especialmente a sexual, enfrentam fragilidades físicas e psicoemocionais que as afastam dos serviços de saúde, dificultando um diagnóstico precoce de ISTs, como a sífilis, ou gerando a descontinuidade do tratamento^(22)^. Ademais, o estigma das ISTs pela sociedade está pautado em julgamentos, estereótipos e atitudes inconscientes que levam ao isolamento e à exclusão social^(23)^.

Desse modo, observa-se que os aspectos culturais e os modos de vida de determinada população influenciam o seu comportamento e as ações e atitudes diante do seu processo de saúde-doença-cuidado. Entre esses aspectos culturais, estão inseridos as crenças, as práticas religiosas, as formas de comunicação e os costumes^(7)^. Gestantes que estão em situação de privação de liberdade e/ou que utilizam substâncias psicoativas (álcool, cigarro e outras drogas) se encontram em uma situação vulnerabilizante e, portanto, têm mais chances de transmissão da doença^(8)^. Diante desses cenários, torna-se essencial avaliar a atitude da gestante em relação ao autocuidado, ao seu estilo de vida, à possiblidade de adesão ou recusa ao tratamento, bem como à qualidade de suas relações interpessoais, familiares e afetivo-sexuais.

A forma como as mulheres constroem seus vínculos afetivos e familiares pode influenciar a forma como lidam com o diagnóstico de sífilis na gestação. Nessa conjuntura, destaca-se a importância das redes e dos suportes sociais, compreendidos como estruturas sociais compostas por pessoas ou organizações conectadas por um ou mais tipos de relações, que compartilham valores e objetivos comuns e oferecem apoio em situações de necessidade dos sujeitos ou da sociedade^(7)^.

Destarte, percebe-se que a VS de gestantes para transmissão vertical da sífilis é uma condição complexa e multifatorial. Entretanto, apresenta-se relacionada a um processo que envolve atitudes e comportamentos que podem ser identificados através de uma ferramenta específica, auxiliando o planejamento de intervenções que contribuam para minimizar o contexto vulnerabilizante no qual as gestantes estão inseridas, prevenindo a sífilis e sua transmissão vertical.

### Limitações do estudo

Destaca-se como limitação do estudo a escassez de estudos que associam a VS à sífilis congênita, estabelecendo o caráter inedito do conteúdo aqui exposto, além da ausência de definições/explicações claras na literatura capazes de conceituar amplamente as DOs que versam sobre as vulnerabilidades do fenômeno estudado. A dificuldade de retorno dos especialistas contatados para o processo de validação das DOs também é constituída como fator limitante.

### Contribuições para a área da enfermagem, saúde ou políticas públicas

Este estudo permite compreender em que dimensões o processo de vulnerabilidade pode se instalar na realidade das gestantes com sífilis, permitindo um olhar mais integro e enfático dos profissionais, em especial os enfermeiros, no cuidado baseado em evidências. Além disso, servirá como arcabouço para posterior elaboração de um instrumento capaz de identificar as VSs das gestantes à transmissão vertical da sífilis nos seus diferentes contextos.

## CONSIDERAÇÕES FINAIS

As definições obtiveram bons índices de evidência de validade. Os ajustes realizados na primeira rodada foram validados pelos juízes na segunda rodada, e as sugestões dos especialistas aperfeiçoaram o instrumento. A dimensionalidade teórica da VS aplicada à transmissão vertical da sífilis é composta por duas dimensões, nove subdimensões e 30 definições constitutivas e operacionais: 22 da dimensão sujeito e oito da dimensão social. São necessários estudos que busquem as evidências de validade do processo de resposta e de estrutura interna do instrumento para confirmar em que medida a dimensionalidade teórica se aplica à população-alvo.

## Data Availability

Os dados de pesquisa disponíveis estão no corpo do artigo.
